# Predicting implementation from organizational readiness for change: a study protocol

**DOI:** 10.1186/1748-5908-6-76

**Published:** 2011-07-22

**Authors:** Christian D Helfrich, Dean Blevins, Jeffrey L Smith, P Adam Kelly, Timothy P Hogan, Hildi Hagedorn, Patricia M Dubbert, Anne E Sales

**Affiliations:** 1 Northwest Health Services Research & Development Center of Excellence, VA Puget Sound Healthcare System, Seattle, Washington, USA; 2 Department of Health Services, University of Washington School of Public Health, Seattle, Washington, USA; 3 Centers for Disease Control and Prevention, National Center for HIV/AIDS, Viral Hepatitis, STD, and TB Prevention, Division of HIV/AIDS Prevention, Atlanta, Georgia, USA; 4 VA Center for Mental Healthcare & Outcomes Research, Arkansas, USA; 5 Research Service, Southeast Louisiana Veterans Health Care Network, New Orleans, Louisiana, USA; 6 VA Mental Health Quality Enhancement Research Initiative, North Little Rock, Arkansas, USA; 7 Center for Management of Complex Chronic Care, eHealth Quality Enhancement Research Initiative, & Spinal Cord Injury Quality Enhancement Research Initiative, Edward Hines, Jr. Veterans Affairs Hospital, Hines, Illinois, USA; 8 Program in Health Services Research, Stritch School of Medicine, Loyola University, Chicago, Illinois, USA; 9 VA Substance Use Disorders Quality Enhancement Research Initiative, Minneapolis VA Healthcare System, Minneapolis, Minnesota, USA; 10 South Central VA Mental Illness Research, Education and Clinical Center (MIRECC), North Little Rock, Arkansas, USA; 11 South Central VA Geriatric Research Education and Clinical Center (GRECC), North Little Rock, Arkansas, USA; 12 VA Inpatient Evaluation Center, Cincinnati, Ohio, USA; 13 VA Health Services Research & Development Center of Excellence, Ann Arbor, Michigan, USA

## Abstract

**Background:**

There is widespread interest in measuring organizational readiness to implement evidence-based practices in clinical care. However, there are a number of challenges to validating organizational measures, including inferential bias arising from the halo effect and method bias - two threats to validity that, while well-documented by organizational scholars, are often ignored in health services research. We describe a protocol to comprehensively assess the psychometric properties of a previously developed survey, the Organizational Readiness to Change Assessment.

**Objectives:**

Our objective is to conduct a comprehensive assessment of the psychometric properties of the Organizational Readiness to Change Assessment incorporating methods specifically to address threats from halo effect and method bias.

**Methods and Design:**

We will conduct three sets of analyses using longitudinal, secondary data from four partner projects, each testing interventions to improve the implementation of an evidence-based clinical practice. Partner projects field the Organizational Readiness to Change Assessment at baseline (n = 208 respondents; 53 facilities), and prospectively assesses the degree to which the evidence-based practice is implemented. We will conduct predictive and concurrent validities using hierarchical linear modeling and multivariate regression, respectively. For predictive validity, the outcome is the change from baseline to follow-up in the use of the evidence-based practice. We will use intra-class correlations derived from hierarchical linear models to assess inter-rater reliability. Two partner projects will also field measures of job satisfaction for convergent and discriminant validity analyses, and will field Organizational Readiness to Change Assessment measures at follow-up for concurrent validity (n = 158 respondents; 33 facilities). Convergent and discriminant validities will test associations between organizational readiness and different aspects of job satisfaction: satisfaction with leadership, which should be highly correlated with readiness, versus satisfaction with salary, which should be less correlated with readiness. Content validity will be assessed using an expert panel and modified Delphi technique.

**Discussion:**

We propose a comprehensive protocol for validating a survey instrument for assessing organizational readiness to change that specifically addresses key threats of bias related to halo effect, method bias and questions of construct validity that often go unexplored in research using measures of organizational constructs.

## Background

There is widespread concern among healthcare systems over gaps in implementing known, evidence-based practices in clinical care [1,2]. There may be as much as a 15 to 20-year lag, on average, before a new evidence-supported practice is integrated into routine care [3]. Evidence suggests that organizations have difficulty systematically implementing new practices, and that the challenge often involves coordinating change among multiple aspects of a practice setting, rather than simply failing to recognize new practices as viable and desirable [1,4-6]. Such complex change initiatives have moderate to poor success rates, with published reviews reporting an approximate 33% median success rate, with much lower success for some sectors [7].

Successful change efforts are characterized by many organizational factors, including employee and manager attitudes about change (to what degree it is possible and desirable); leadership support (making the change a priority); slack resources; adequate planning (clarity of goals and roles); and mechanisms for tracking and reporting progress. Some organizational scholars propose that these factors are generally observable at the outset of a change initiative, and taken collectively, constitute an organization's readiness to make the change [8-10]. If accurately assessed, baseline organizational readiness could be used prognostically to predict the likelihood of successful change or diagnostically for formative evaluation. Many surveys have been published to measure organizational readiness [9,10]. However, few have undergone rigorous validation, notably to demonstrate the ability to prospectively distinguish successful change efforts from those that will fail [9,10].

In this paper, we briefly review literature on measures of organizational readiness for change (ORC) and discuss three specific threats that pose challenges for validating measures of organizational readiness [11-13]. Next, we describe our protocol for validation of a previously developed instrument, the Organizational Readiness for Change Assessment (ORCA) [14], and how we address key threats to validity.

### Background and literature review: What we currently know about organizational readiness to change

We define organizational change as planning and actions to alter collective behavior in the pursuit of specific objectives [15], notably the implementation of evidence-based clinical practice. Examples may include implementation of a best-practices bundle for cardiovascular disease risk management [16], or a collaborative care model for treating depression in primary care [17]. Researchers frequently observe different levels of preparedness among organizations adopting the same evidence-based practice [8,10]. This psychological, behavioral, and structural preparedness is what we refer to as ORC. The proximal outcome of ORC should be implementation effectiveness, meaning how effectively a clinical practice change is made [18]. This is different than measuring how effective the practice change ultimately is on care provision, which we refer to as innovation effectiveness [18], arguably affecting more distal outcomes (*e.g*., improving patient satisfaction, quality of care, efficiency or patient outcomes).

Two recent systematic literature reviews have examined tools for measuring ORC [9,10]. A 2008 systematic review found 103 published peer-reviewed papers addressing organizational readiness, the majority being empirical studies, with 53 concerning healthcare settings [10]. They report outcomes such as increasing levels of patient engagement with substance-abuse treatment [19]; successful implementation of varied health service programs by hospitals [20]; quality improvements for cardiac surgery programs [21]; and adoption of evidence-based treatment practices [22]. These studies have often reported very large effect sizes, such as an R^2 ^of 0.47 for predicting short-term implementation of quality improvements for cardiac surgery programs [21], and an area under the receiver operator characteristic (ROC) curve in excess of 0.84 for distinguishing successful from unsuccessful implementation of change efforts reported by hospital executives [20].

However, this research has relied almost exclusively on instruments that have little or no published information about their psychometric properties [9,10]. Where validation analyses have been conducted, findings have often been ambiguous or methodologically flawed. For example, studies linking ORCA to outcomes often used self-reported outcomes and measured both ORC and outcomes after the fact [20,21], which as we explain below introduces bias. In the most recent review, Weiner and colleagues identified 43 unique instruments for measuring ORC [10]. Seven of these instruments, summarized in Table 1, were both available in the public domain and had undergone systematic assessment of psychometric properties, including scale reliability, and construct, content, and criterion validities [19,23-28]. Yet, each of the seven had further deficits that limit their utility as a standard measure for studying the determinants of organizational change [10].

**Table 1:** ORC instruments with published psychometrics and validation issues

ORC instrument	Description	Validation issue	Key citations
Organizational e-readiness	Measures organizational members' perceptions of readiness for adoption of e-commerce.	Not suited to measuring implementation of general, evidence-based health service practices.	[27,79]

Organizational readiness	Measures organizational members' perceptions of organization's data warehouse process maturity.	Not suited to measuring implementation of general, evidence-based health service practices.	[28]

Organizational readiness for change	Two scales drawn from Pasmore Sociotechnical Systems Assessment Survey (STSAS) measuring innovativeness and cooperativeness.	The STSAS, while validated, was not designed or validated to be a measure of ORC; authors drew on two subscales they believed are related to organizational readiness.	[24]

TCU organizational readiness for change	Measures organizational members' perceptions of the motivation for change, adequacy of resources, staff attributes, and organizational climate.	Extensively used, with published evidence of reliability and validity. However, results have varied, with poor scale reliability reported by recent studies, and inconsistent relationships observed between individual scales or readiness dimensions and outcomes.	[19,22,80,81]

Change-related commitment	Measures employee's agreement and willingness to work toward a goal of organizational change.	Published evidence of reliability and validity, but designed for individual-level factors. Ignores the role of interdependence among the individuals involved.	[23]

Commitment to change	Measures three dimensions of organizational members' commitment to a change: affective commitment, continuance commitment, and normative commitment.	Published evidence of reliability and validity, but designed for individual-level factors. Ignores the role of interdependence among the individuals involved.	[25]

Readiness for organizational change	Measures organizational members' perceptions of the appropriateness of change, management support, self-efficacy and personal benefit.	Published evidence of reliability and validity, but designed for individual-level factors. Ignores the role of interdependence among the individuals involved.	[26]

Summarized from Weiner BJ, Amick H, Lee S-YD: **Conceptualization and measurement of organizational readiness for change: A review of the literature in health services research and other fields**. *Medical Care Research and Review *2008, **65(4):**379-436.

### Issues in establishing psychometric properties of ORC instruments

There are a range of widely-recognized criteria for psychometric validation of survey instruments [29,30]. In particular, there are three psychometric tests that we propose are of special importance or pose unique challenges for validating organizational construct measures: inter-rater agreement, predictive validation, and discriminant validation.

First, it is critical to assess the level of shared perception in a collective phenomenon, such as organizational readiness. If individuals fail to share the same perception, then it can be argued that the phenomenon is not organizational [31]. For this reason, organizational scholars propose four minimum criteria for aggregating individual survey data into collective units (*e.g*., teams or facilities): a theoretical rationale that the phenomenon is collective; appropriate item structure (*i.e*., items written in the perspective of the collective as opposed to the individual); demonstration of adequate reliability of the scale at the team-level; and adequate inter-rater agreement [31].

Second, predictive validity is the degree to which a measure accurately predicts some outcome of interest (*e.g*., objective changes in behavior). While predictive validity is generally the *sine qua non *of survey validation [15,32], research designs for predictive validation vary widely, and some frequently used methods may introduce threats to validity. In some studies, respondents retrospectively answer questions about organizational factors (*i.e*., the independent variables) and change outcomes (*i.e*., dependent variable) with the same instrument at the same point in time [20,21,33,34], potentially introducing common method bias. Common method bias encompasses a range of biases, such as recall bias and halo effect, that can produce spurious associations or grossly inflate true associations [35]. Researchers disagree about the extent to which common method variance biases results, but estimates suggest it accounts for 18% to 26% of the observed variance in constructs measured [36,37].

Finally, discriminant validity is 'the degree to which the measure is not similar to (diverges from) other measures that it theoretically should not be similar to' [35]. Discriminant validity is particularly important in psychometric validation of organizational surveys because of bias from the 'halo effect,' a human tendency to infer specific attributes about a person or entity from one's overall impressions [11]. Halo effect has been shown to produce Pearson correlations of 0.47 to 0.91 among very disparate constructs [38], and experiments have artificially induced a halo effect in team members' evaluation of team dynamics by manipulating information about their performance [39].

In the context of measuring ORC, our concern is that a halo effect could arise from knowing the outcome of the change, or from overall feelings toward the organization such as job morale or relationship quality with supervisors. In the latter case, the source of halo effect (*e.g*., job morale) may share a common cause with the performance outcome being measured, and therefore introduce confounding even for prospective criterion validation studies.

### The organizational readiness for change assessment (ORCA)

In the funded study described in this protocol, we are using an ORC instrument developed by members of the study team, called the ORCA. The ORCA was initially developed by researchers in the Ischemic Heart Disease Quality Enhancement Research Initiative (IHD QUERI), part of a larger national initiative in the United States Department of Veterans Affairs Office of Research and Development. The original purpose of the ORCA was to assess organizational-level variables that were posited to influence implementation of evidence-based clinical practice, focusing on specific practice innovations, such as increasing lipid measurement and management in ischemic heart disease. It has been used as part of several evidence-based practice implementation efforts in the Veterans Health Administration (VA).

The ORCA (Additional File 1) is a structured survey intended to assess organizational readiness to implement a specific, evidence-based clinical practice. It is intended to provide an overall indication of the likelihood of success at baseline, and to assess changes over time.

**Figure 1:**
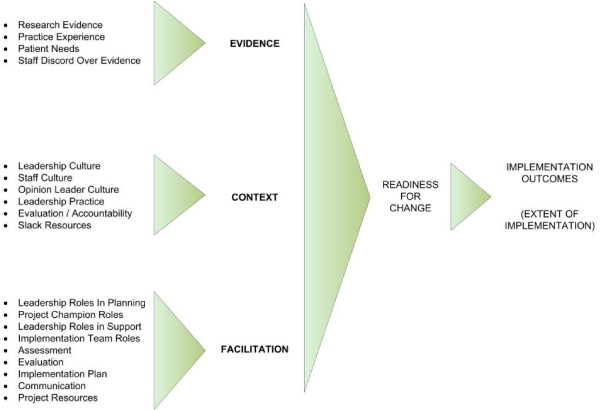
**ORCA scales, subscales and outcomes**. This figure illustrates the composition of the ORCA scales and their hypothesized relationship to organizational readiness for change, and subsequently to implementation outcomes.

Figure 1 depicts the three primary scales and 19 subscales comprising the ORCA. The survey is meant to be filled out by clinical and administrative staff involved in implementation of the evidence-based practice, particularly members of teams charged with evidence-based practice implementation. The survey is anchored to the specific change by an opening statement about what the practice change is expected to achieve, *e.g*., 'the ICU infection control bundle at [facility x] will reduce nosocomial infections among ICU patients.'

A detailed description of the instrument and results from scale reliability and factor structure analyses have been previously published [14], and colleagues have reported findings that suggest the instrument may be effective in predicting implementation outcomes [40]. However, the instrument has not been comprehensively validated.

### Objectives of the study protocol

The objective of our study protocol is to conduct a comprehensive assessment of the psychometric properties of the ORCA. Our primary aims are to:

1. Extend current knowledge about the ORCA's measurement reliability, as indicated by meeting or exceeding minimum thresholds for assessing inter-rater, and internal consistency reliabilities.

2. Extend current knowledge about the ORCA's content validity, particularly within VA, using a modified Delphi technique with recognized VA and non-VA experts in organizational change, and empirically matching ORCA items and subscales to theoretical content domains.

3. Assess four types of criterion validity for the ORCA: predictive, concurrent, convergent, and discriminant validities.

## Methods

### Data and settings

Data will be aggregated from four intervention studies designed to implement evidence-based practice changes in clinical settings within the VA. These partner projects are described in detail in Additional File 2[41-71]. We are collaborating with each partner project to ensure the collection of equivalent data on important organizational dimensions to allow us to aggregate across samples. These include how implementation outcomes are measured, and the timeframe in which ORCA and implementation outcomes are being measured.

In each partner project, the ORCA is administered prospectively to providers and staff from each VA medical center or community-based outpatient clinic site participating in the implementation of the evidence-based practice. Each partner project determines their timeline for baseline-survey collection to ensure respondents are aware of the planned practice changes and can meaningfully participate in the survey before implementation activities are completed.

All four partner projects test the effects of an external facilitation intervention on the implementation of an evidence-based practice. External facilitation is a process of interactive problem-solving and support by individuals or teams that are external to the organization implementing the innovation [71]. It uses multiple techniques and evolves in response to variable site characteristics, resources, and barriers.

Implementation outcomes are measured between six and nine months following baseline administration of the ORCA and initiation of external facilitation. Each partner project determines timing of outcome and follow-up measures to ensure adequate time for practice changes to occur and to provide measurement at equivalent timeframes across all studies. Partner projects collect outcome data as the proportion of users that have implemented the practice change, or the proportion of cases where the practice change occurred. This will allow us to standardize outcomes as an effect size and to analyze pooled data.

Two of the partner projects are also administering the ORCA at their follow-up assessment six to nine months following baseline, and fielding additional job satisfaction items for convergent and discriminant validity analyses.

The VA's Central Institutional Review Board (CIRB) deemed this study exempt from the standard human subjects ethical research requirements.

### Analyses

To meet our objective to comprehensively assess the psychometric properties of the ORCA, we will conduct three sets of psychometric analyses corresponding to our three study aims: two scale and item reliability analyses; content validity analyses; and four criterion validity analyses. These are summarized in Table 2.

**Table 2:** Overview of validation analyses for primary aims

Type of validation	Definition	Analysis	Data Source	Observations
Aim 1				

Inter-rater reliability	The consistency of measurement results across different raters given identical conditions	ICC calculated from HLM to determine if respondents have higher agreement within facility and project than between.	Individual-level, baseline ORCA data from partner projects	k = 208 n = 53

Internal consistency reliability	The consistency of items within a given scale, with the same rater	Cronbach's alpha, and item-rest correlation to determine if items within subscales, and subscales within scales, correlate more strongly than between subscales/scales.	Individual-level, baseline ORCA data from partner projects	k = 208 n = 53

Aim 2				

Content validity	A check of the instrument's items against the content domain of the construct	Expert panel review of conceptual domains, and Delphi survey on ORCA items assessing (a) degree of match to conceptual domain, and (b) importance for understanding organizational readiness;	Transcripts of expert panel discussion and structured Delphi survey	n = 14 (panel members)
		
		Confirmatory factor analysis to match items to subscales, and subscales to scales.	Individual-level, baseline ORCA data from partner projects	k = 208 n = 53

Aim 3				

Predictive validity	Degree to which an instrument predicts a theoretically meaningful outcome.	Multivariate regression in which the ORCA scales serves as IV, and implementation effect size as the DV.	Site-level, baseline ORCA data, and individual-level implementation outcomes	k = 146 n = 30

Concurrent validity	Degree to which an instrument distinguishes groups it should theoretically distinguish (*e.g*., low false positives and low false negatives).	Multivariate regression in which external facilitation intervention is the IV and the ORCA scales are the DV.	Site-level, follow-up ORCA data, and intervention cohort (external facilitation vs. control site)	k = 122 n = 28

Convergent validity	The degree to which an instrument performs in a similar manner to other instruments that purportedly measure the same construct.	Multivariate regression with ORCA scales as IVs, and JSI items on satisfaction with direct supervision and senior leadership serve as DVs.	Individual-level, baseline ORCA and job satisfaction data	k = 158 n = 33

Discriminant validity	Degree to which an instrument performs in a different manner to other instruments that measure different constructs.	Multivariate regression with ORCA scales as IVs, and overall JSI and satisfaction with pay as DVs.	Individual-level, baseline ORCA and job satisfaction data	k = 158 n = 33

IV = independent variable, DV = dependent variable, ORCA = Organizational Readiness for change Assessment, JSI = job satisfaction index, HLM = Hierarchical Linear Modeling, k = number of individual respondents, n = number of sites.

We propose to conduct analyses at two levels. First, item-scale reliability analyses, confirmatory factor analysis (for content validation), and convergent and discriminant validity analyses will use individual-level data from the ORCA. As explained in more detail below, the reliability and factor analyses are based on correlations among items within respondents, and on correlations among respondents within facilities. Second, the inter-rater reliability analyses, the predictive validity, and concurrent validity analyses will be at the facility-level, examining differences within and between facilities on aggregated ORCA scales and implementation outcomes.

ORCA scores will be tallied for each of the three scales at the facility level as the average of respondents' scores. The scores for each respondent will be tallied as the average of the constituent subscale scores. The average of subscales is used instead of the average of items because subscales are of different lengths, and calculating the average of the items would give relatively higher weight to longer subscales. ORCA scores will be treated as linear, continuous variables.

### Scale and item reliability analyses (aim one)

We will conduct two assessments of reliability. First, we will assess inter-rater reliability, which poses a challenge for organizational measures because raters do not overlap organizations (*i.e*., raters do not serve in multiple organizations and rate each one). It is possible to attribute variation in response to raters within an organization, but not to raters between organizations. This makes traditional measures such as Cohen's or Fleiss' kappa inappropriate [72]. A solution is to use an approach that considers the nested nature of the data (multiple raters within each organization). We will use hierarchical linear modeling (HLM), employing an empty model to separately estimate variance in ORCA scale scores that is due to the rater, versus the organization. The reliability coefficient is calculated from the variance estimates as the intra-class correlation (ICC), which is the proportion of total variance that is attributable to disagreements among raters. To the extent that raters agree, then rater-level variation is low, and the ICC will be high. This procedure requires multiple raters for some observations, but can accommodate different numbers of raters per organization [72]. Inter-rater reliability will be assessed using data from all four partner projects. We will test for significant differences in mean reliability coefficients among the three ORCA scales from partner projects using z-tests. An additional level of nesting is present in the data: organizations are nested within each of the four studies. The HLM approach will also examine how much of the variation in ORCA score across sites can be attributed to each of the partner projects providing data.

Second, internal-consistency reliability is the extent to which items from the same hypothetical scale or subscale correlate with each other as predicted. This is an important assessment prior to aggregating survey items into subscales and scales [35]. These analyses will be done in two stages: first focusing on the subscales and secondly on the scales. Internal consistency reliability will be assessed with two measures of item correlation with a given subscale:

(1) Cronbach's alpha is a summary measure of the average correlation among all possible combinations of items divided into equal pools. It provides a rough estimate of the cohesiveness of a set of items. We will assess the effect on the Cronbach's alpha of eliminating any one item from its given subscale to help identify specific items that contribute to poor reliability. (2) Item-rest correlation is the correlation of a given item to the remaining items collectively in its hypothesized scale or subscale, and is an indicator of the cohesiveness of the specific item with its corresponding scale. It is another method to help identify specific items that contribute to poor reliability [73]. Cronbach's alpha is a scale-level measure of reliability, and item-rest correlation is an item-level measure of reliability [73]. For the second stage, we will calculate the Cronbach's alpha for the overall scales (*e.g*., the evidence scale) as a function of the constituent subscales (*i.e*., the aggregated subscale scores). Subscales or items that contribute to poor scale reliability may be dropped from validity analyses, and be used to develop a shortened-form of the survey (aim five). These analyses are based on correlations among items within-respondent, and thus should not be a function of a specific setting or organizational change [73]. For this reason, observations across the partner projects will be pooled for the internal-consistency reliability analyses. Where a follow-up ORCA assessment is conducted and more than one observation exists for an individual, the first observation will be used. We will adhere to published recommendations for handling missing data [30].

### Content validity assessment (aim two)

Content validity is the extent to which items in a measure represent the content of interest within the conceptual domain. Assessment of content validity can be accomplished through matching of item content to specific units of a textual representation of the content domain and/or expert opinion that such matching exists and is adequate [32]. For ORCA, we propose to: trace each of the 77 items to their corresponding subscales) and report on the status of matches using confirmatory factor analysis (CFA); and convene an expert panel via conference calls to elaborate critical domains for understanding ORC, and use a modified Delphi technique among a second group of experts to rate the adequacy of the ORCA's content coverage of those domains [74].

For the first step, we will use CFA to trace the items back to content domains. Weiner *et al*. recommend factor analysis as an indicator of content validity for multidimensional constructs because it can be used to verify the existence of the theorized dimensions [10]. We will use CFA to assess the fit between data from the partner projects and the 19 subscales of the ORCA. Following recommendations from Joreskog and Sorbom, we will begin by tracing a single latent variable to its corresponding observed variables (*i.e*., the items comprising an individual subscale), then proceed to simultaneously test pairs of factors, and finally to testing the combination of factors comprising each scale [75].

For the second step, the expert panel described earlier will participate in a roundtable discussion via conference call to discuss and identify the conceptual domains and dimensions critical for understanding ORC. The conference call will be transcribed verbatim, and coded for consensus conceptual domains critical for understanding ORC. Summaries of the coded domains will be distributed via e-mail to expert panel members for comment and revision.

A second, larger group of experts, which may include some participants from the expert panel, will participate in a modified Delphi process via e-mail to match and rate ORCA items and the expert-panel derived domains. The Delphi technique is an established method for 'forming consensus and defining levels of agreement about issues of uncertainty among groups of individuals who are separated by time and space' [76]. After reviewing the items and matched content, Delphi members will assign each item two scores: a score from 1 (lowest) to 10 (highest) representing the importance of the item for understanding ORC; and a categorical assessment of which conceptual domain it matches. Members will also be asked to comment on the readability and accuracy of any items they find problematic. The investigators will merge the results and provide the Delphi members the following for each item: their own scores previously assigned; the Delphi panel median scores; the panel twenty-fifth and seventy-fifth percentiles; and a de-identified list of comments on the item. Members will then use this information to repeat the scoring process, free to either keep their previous scores or change their scores, and provide additional comments if desired. Those who score an item outside the twenty-fifth or seventy-fifth percentile will be asked to provide a written reason for their score. This scoring and feedback cycle will be performed up to three times; if there are fewer than 10% changes on the second round, we will not repeat the process. The results will be presented to Delphi members, and a final opportunity to make written comments on items will be provided. The final product will be an item-by-item assessment of the content validity of the ORCA vis-à-vis the expert panel-derived domains. A major advantage of the modified Delphi technique is the ability to generate high-quality consensus without the need for a physical meeting.

### Criterion validity analyses (aim three)

Predictive Validity is the extent to which the measure predicts a theoretically meaningful outcome [35]. Unlike reliability analyses, which assess correlations among items within respondent, or among respondents within the facility, the criterion analyses are at the site level. For ORCA, the outcome we wish to predict is the extent of implementation, which we term 'implementation outcome.' Psychometric assessment of predictive validity is concerned with the specific issue of establishing whether a relationship exists between the instrument and a relevant outcome. For example, an IQ test might be expected to predict subsequent school grades.

To test the predictive validity of the ORCA, we will conduct HLM. The dependent variable is implementation outcome measured as an effect size. The partner projects will measure implementation outcome as a proportion of care practices changed, measured at the site level or at the provider-level and aggregated to the site level (described in Additional File 2), which will be transformed into an effect size based on change from baseline to follow-up. For example, one partner project sought to increase the use of cognitive behavioral therapy for depression; the outcome of interest is the change from baseline to follow-up in the percent of clinic time over the past 30 days that therapists report using cognitive behavioral therapy to treat depression [43]. We will convert change in proportions across all four projects into a single standardized effect size measure, Cohen's h [44]. Cohen's h employs an arcsine transformation of the proportion scores, which standardizes differences between proportions at any given magnitude of those proportions. This provides a standardized outcome that can be analyzed in aggregate.

Independent variables will include partner project sample (four categories represented by three dummy coded variables), and whether the site received the external facilitation intervention as part of the partner project or was a comparison site (two categories represented by one dummy coded variable). ORCA scores will be entered into the equation as continuous variables.

We will conduct a secondary analysis to quantify the size of the relationship between the ORCA and implementation outcomes.

Concurrent validity is the extent to which the measure is able to distinguish between groups that should theoretically differ [35]. In the context of the ORCA, an important indication of concurrent validity will be distinguishing the facilities in the partner projects that receive external facilitation activities (intervention sites) from those receiving none (control sites)[71]. The external facilitation intervention, if it is effective, should alter scores on the ORCA, particularly the facilitation scale, over time. In the present study, we will assess changes in ORCA scores from baseline to follow-up between sites receiving external facilitation (n = 14) and control sites (n = 14). We will test the hypothesis that the change in ORCA scores is positive and larger (meaning greater readiness for change) among facilitation sites relative to control sites. In the predictive validity analyses, we expect at least 30 observations (*i.e*., at least 30 sites). Data for 20 of the sites have been collected. The remaining sites come from one partner project currently in start-up at 12 sites; in calculating our power, we have conservatively allowed for the attrition of two of those sites. With 30 observations, we will have 90% power to detect an effect of ORCA score that is equal to or greater than R^2 ^= 0.21 (with type I error rate set to 0.05, two tailed) [44]. We will have 80% power to detect an effect of ORCA score that is equal to or greater than R^2 ^= 0.17 (with type I error rate set to 0.05, two tailed). This power calculation conservatively estimates that the other predictors (study sample and external facilitation) will account for no more than 15% of the variability in implementation effect.

### Convergent and discriminant validities

Convergent validity is the extent to which the measure converges on other measures that it theoretically should be similar to--most often other measures of the same or related constructs [35]. The challenge to assessing convergent validity is that we are interested in validating the ORCA precisely because systematic reviews conclude there is a dearth of well-validated instruments [9,10]. Thus, as detailed below, we chose the best measures of similar and dissimilar constructs possible.

Discriminant validity is particularly salient in measuring multi-dimensional constructs, such as ORC (19 distinct subscales in the ORCA), because such constructs are inherently broad and complex; thus, we would expect them to correlate with many related organizational measurements (*e.g*., organizational culture). To test convergent and discriminant validities, we will compare ORCA scales to employee morale as measured by the Job Satisfaction Index (JSI) (Appendix B). The JSI is a validated, 12-item short-form [77] of the Job Descriptive Index scale which measures five dimensions of satisfaction with work in addition to overall satisfaction: the work itself, coworkers, management and leadership, opportunities for promotion, and pay [65]. The JSI has a track record of use in VHA, and is fielded annually in the All Employee Survey. We hypothesize that ORC may be related to job satisfaction; organizations that are better prepared to effectively implement change may be more satisfying places to work [10]. However, we should observe different relationships between ORC and particular dimensions of job satisfaction, and these different relationships with dimensions of job satisfaction provide a compelling test of convergent and discriminant validities. For example, several of the ORCA subscales assess roles and characteristics of organizational leadership. Therefore, we would expect ORCA scores to have a strong, positive correlation (R^2 ^≥ 0.20) to JSI measures of satisfaction with management and leadership. To test this hypothesis, we will build separate regression models, with the three ORCA scales predicting JSI satisfaction with management and leadership. As before, we will have sufficient power to detect medium-sized (R^2 ^= 0.15) or larger effects.

Conversely, level of employee pay is largely prescribed by General Schedule pay tables for federal employees, occupation and tenure, and is an individual-level variable, not an organizational-level one. Therefore we expect little or no significant association (R^2 ^≤ 0.10) between ORCA and a JSI measure of satisfaction with pay. If the ORCA scales, particularly context, have equally strong correlations with measures of satisfaction with leadership and pay, it suggests that respondents may be inferring answers to ORCA items from their overall feelings of satisfaction with their work.

Overall job satisfaction will be a function of satisfaction with pay, leadership, and a range of other factors, such as the work itself and relationships with coworkers [65], which may be correlated with ORC, but should not be as strongly correlated as satisfaction with leadership, which are dimensions specifically measured in the ORCA. Therefore we hypothesize that ORC will have a significant but moderate relationship (R^2 ^= 0.10 to 0.20) with overall job satisfaction. In sum, we expect to see the largest relationship between ORCA scales and satisfaction with direct supervision and senior leadership, and the smallest relationship to satisfaction with pay, with the relationship to overall job satisfaction falling somewhere in between.

## Discussion

The proposed study will conduct a battery of psychometric validation analyses on a promising survey instrument to assess ORC. The protocol focuses on three psychometric practices that we argue pose particular challenges for validation of measures of organizational constructs, or are rarely completed: inter-rater agreement, predictive validation using prospective data, and convergent and discriminant validation. By conducting this research, we address a noted gap in the literature [9,10,13], and contribute to a stronger scientific base for implementation research.

## Potential limitations

The proposed study has two limitations. The first limitation is our reliance on aggregated data from four partner projects. It introduces potential challenges to both analyses and study management. The partner projects may contribute non-equivalent data resulting from either differences in data collection methods or fundamental differences in the study samples. To mitigate this threat, we engaged partner projects in the earliest stages of design of the proposed study, and recruited the PIs of the partner projects to serve as co-investigators on the proposed validation study. This included multiple conversations to ensure familiarity with the specifics of the partner projects, including the ORCA administration procedures, uses of the ORCA data, and challenges encountered. As a result, we were able to ensure a level of comparability of study measurements and outcomes that would not be possible by simply aggregating secondary data.

At the same time, capitalizing on data from multiple, real-work implementation projects has some advantages. By partnering with existing and planned implementation projects, the proposed study will validate the ORCA against real, not hypothetical implementation outcomes. Using prospective, real-world data increases our confidence that positive findings will not be the result of a spurious halo effect, and consequently that the findings will be applicable to those doing implementation work.

In addition, pooling data from multiple studies likely produces more generalizable results owing to the diversity of the partner projects. By design, this study encompasses multiple implementation projects, and avoids the threat that reliability and validity findings are unique to a specific change, set of actors, or setting, that would make them non-generalizable to other settings or populations.

The second limitation is the sample size, which will be small relative to retrospective study designs and validation studies that are respondent level and not organizational level. A small sample poses particular challenges for criterion validation. While larger samples are, all things being equal, preferable, the central issue is what is necessary to infer criterion validity. A larger sample would be necessary to account for small (but statistically significant) variance in our proposed models. However, for the ORCA to be of value operationally to the VA, a large relationship is needed. If the ORCA fails to account for at least 15% of the variation in implementation (the level we set in our power calculations) in a relatively simple model, we argue that it is unlikely to be operationally useful. Accounting for small amounts of variance, while of interest academically, will not be useful to decision making in how to better engage in the implementation of evidence-based programs.

We briefly also note a methodological choice about the basic psychometric approach we propose. These analyses represent a classical test-theory approach, whereas much contemporary psychometric work is based on item response theory. We propose a classical test-theory approach because most applications of item response theory focus on unidimensional scales and address research goals such as identification of items that are subject to group biases, or creation of banks of items that can be used in adaptive testing. Given that our objective is to create a single measure comprising multiple dimensions, item response theory methods add complexity without providing an advantage over a classical approach [78].

## Conclusions

In this paper, we propose a comprehensive protocol for validating a survey instrument for assessing ORC. This protocol specifically addresses key threats of bias related to halo effect, method bias, and questions of construct validity that often go unexplored in research using measures of organizational constructs. The methods presented in this protocol are broadly applicable to validation of surveys to measure other organizational constructs, such as organizational culture, climate for safety, and team functioning. We believe this protocol can serve as a survey validation model for a range of organizational constructs.

## Competing interests

The authors declare that they have no competing interests.

## Authors' contributions

CDH is the principal investigator for this funded study; DB, PAK, JLS, TPH, HH, and PMD are co-investigators, and AES is a key collaborator. CDH took the lead in drafting the text; all authors critically reviewed it and contributed to the study proposal on which it is based. All authors read and approved the final manuscript.

## Supplementary Material

Additional file 1 **Copy of the Organizational Readiness to Change Assessment instrument**. This file is a PDF format of the Organizational Readiness to Change Assessment instrument with annotations about where the instrument is to be customized. Click here for file

Additional file 2 **Description of four partner projects**. This file is a PDF document describing each of the four partner projects contributing data to the study for the described protocol, including the project aims, methods and details about the use of the ORCA. Click here for file

## References

[B1] Berwick DM Disseminating innovations in health care Jama 2003 289 15 1969 75 10.1001/jama.289.15.1969 12697800

[B2] Institute of Medicine Committee on Quality of Health Care in America Crossing the quality chasm: a new health system for the 21st century 2001 Washington, D.C.: National Academy Press

[B3] Balas EA Boren SA Medicine NLo. Bethesda MD Managing clinical knowledge for health care improvement. Yearbook of Medical Informatics 2000 65 70 27699347

[B4] Dopson S Locock L Chambers D Gabbay J Implementation of evidence-based medicine: evaluation of the Promoting Action on Clinical Effectiveness programme Journal of Health Services Research and Policy 2001 6 23 31 10.1258/1355819011927161 11219356

[B5] Weiner BJ Savitz LA Bernard S Pucci LG How do integrated delivery systems adopt and implement clinical information systems? Health Care Management Review 2004 29 1 51 66 14992484 10.1097/00004010-200401000-00007

[B6] Nutting PA Miller WL Crabtree BF Jaen CR Stewart EE Stange KC Initial Lessons From the First National Demonstration Project on Practice Transformation to a Patient-Centered Medical Home Ann Fam Med 2009 7 3 254 260 10.1370/afm.1002 19433844 PMC2682981

[B7] Smith ME Success rates for different types *of *organizational change Performance Improvement 2002 41 1 26 33 10.1002/pfi.4140410107

[B8] Armenakis AA Harris SG Mossholder KW Creating Readiness for Organizational Change Human Relations 1993 46 6 681 703 10.1177/001872679304600601

[B9] Holt D Armenakis A Harris S Feild H Toward a comprehensive definition of readiness for change: a review of research and instrumentation Research in Organizational Change and Development 2006 JAI Press: Amsterdam, Netherlands

[B10] Weiner BJ Amick H Lee SYD Conceptualization and Measurement of Organizational Readiness for Change: A Review of the Literature in Health Services Research and Other Fields Med Care Res Rev 2008 65 4 379 436 10.1177/1077558708317802 18511812

[B11] Rosenzweig P Misunderstanding the Nature of Company Performance: The Halo Effect and Other Business Delusions California Management Review 2007 49 4 6 20

[B12] Spector PE Method Variance in Organizational Research Organizational Research Methods 2006 9 2 221 232 10.1177/1094428105284955

[B13] Hinkin TR A Review of Scale Development Practices in the Study of Organizations Journal of Management 1995 21 5 967 988

[B14] Helfrich C Li Y-F Sharp N Sales A Organizational readiness to change assessment (ORCA): Development of an instrument based on the Promoting Action on Research in Health Services (PARIHS) framework Implementation Science 2009 4 1 38 10.1186/1748-5908-4-38 19594942 PMC2716295

[B15] Weiner BJ Amick H Lee SYD Conceptualization and measurement of organizational readiness for change: A review of the literature in health services research and other fields Medical Care Research and Review 2008 65 4 379 436 10.1177/1077558708317802 18511812

[B16] Scott SD Plotnikoff RC Karunamuni N Bize R Rodgers W Factors influencing the adoption of an innovation: An examination of the uptake of the Canadian Heart Health Kit (HHK) Implement Sci 2008 3 1 41 10.1186/1748-5908-3-41 18831766 PMC2567341

[B17] Hedrick SC Chaney EF Felker B Liu CF Hasenberg N Heagerty P Buchanan J Bagala R Greenberg D Paden G Fihn SD Katon W Effectiveness of collaborative care depression treatment in Veterans' Affairs primary care J Gen Intern Med 2003 18 1 9 16 10.1046/j.1525-1497.2003.11109.x 12534758 PMC1494801

[B18] Klein K Sorra J The challenge of innovation implementation Academy of Management Review 1996 21 4 1055 1080

[B19] Lehman WEK Greener JM Simpson DD Assessing organizational readiness for change Journal of Substance Abuse Treatment 2002 22 4 197 209 10.1016/S0740-5472(02)00233-7 12072164

[B20] Gustafson DH Sainfort F Eichler M Adams L Bisognano M Steudel H Developing and Testing a Model to Predict Outcomes of Organizational Change Health Services Research 2003 38 2 751 776 10.1111/1475-6773.00143 12785571 PMC1360903

[B21] Molfenter T Gustafson D Kilo C Bhattacharya A Olsson J Prospective evaluation of a Bayesian model to predict organizational change Health Care Manage Rev 2005 30 3 270 9 16093893 10.1097/00004010-200507000-00011

[B22] Fuller BE Rieckmann T Nunes EV Miller M Arfken C Edmundson E McCarty D Organizational Readiness for Change and opinions toward treatment innovations Journal of Substance Abuse Treatment 2007 33 2 183 192 10.1016/j.jsat.2006.12.026 17434708 PMC2031859

[B23] Jansen KJ From persistence to pursuit: A longitudinal examination of momentum during the early stages of strategic change Organization Science 2004 15 3 276 294 10.1287/orsc.1040.0064

[B24] Ingersoll GL Kirsch JC Merk SE Lightfoot J Relationship of Organizational Culture and Readiness for Change to Employee Commitment to the Organization Journal of Nursing Administration 2000 30 1 11 20 10.1097/00005110-200001000-00004 10650431

[B25] Herscovitch L Meyer JP Commitment to organizational change: Extension of a three-component model Journal of Applied Psychology 2002 87 3 474 487 12090605 10.1037/0021-9010.87.3.474

[B26] Holt DT Armenakis AA Feild HS Harris SG Readiness for Organizational Change: The Systematic Development of a Scale Journal of Applied Behavioral Science 2007 43 2 232 255 10.1177/0021886306295295

[B27] Molla A Licker PS eCommerce adoption in developing countries: a model and instrument Information & Management 2005 42 6 877 899 10.1016/j.im.2004.09.00221786449

[B28] Sen A Sinha AP Ramamurthy K Data warehousing process maturity: An exploratory study of factors influencing user perceptions IEEE Transactions on Engineering Management 2006 53 3 440 455

[B29] Scientific Advisory Committee of the Medical Outcomes Trust Assessing Health Status and Quality-of-Life Instruments: Attributes and Review Criteria Quality of Life Research 2002 11 3 193 205 10.1023/A:1015291021312 12074258

[B30] Nunnally JC Bernstein IH Psychometric Theory 1994 New York, NY: McGraw-Hill Inc.

[B31] Tesluk P Mathieu JE Zaccaro SJ Marks M Brannick MT, Salas E, Prince C Task and aggregation issues in the analysis and assessment of team performance, in Team performance assessment and measurement: theory, methods, and applications 1997 Lawrence Erlbaum Associates: Mahwah, N.J 197 224

[B32] Hinkin TR A Brief Tutorial on the Development of Measures for Use in Survey Questionnaires Organizational Research Methods 1998 1 1 104 121 10.1177/109442819800100106

[B33] Bahtsevani C Willman A Khalaf A Östman M Developing an instrument for evaluating implementation of clinical practice guidelines: a test-retest study Journal of Evaluation in Clinical Practice 2008 9999 9999 10.1111/j.1365-2753.2007.00916.x 18331325

[B34] Cummings GG Estabrooks CA Midodzi WK Wallin L Hayduk L Influence of organizational characteristics and context on research utilization Nurs Res 2007 56 4 Suppl S24 39 17625471 10.1097/01.NNR.0000280629.63654.95

[B35] Trochim WMK The Research Methods Knowledge Base 2000 Atomic Dog Publishing.com

[B36] Lance CE Dawson B Birkelbach D Hoffman BJ Method Effects, Measurement Error, and Substantive Conclusions Organizational Research Methods 2010 13 3 435 455 10.1177/1094428109352528

[B37] Podsakoff P MacKenzie S Lee J Podsakoff N Common method biases in behavioral research: A critical review of the literature and recommended remedies Journal of Applied Psychology 2003 88 5 879 14516251 10.1037/0021-9010.88.5.879

[B38] Thorndike EL A constant error in psychological ratings Journal of Applied Psychology 1920 25 29

[B39] Staw BM Attribution of the "causes" of performance: a general alternative interpretation of cross-sectional research on organizations 1974 [Urbana]: College of Commerce and Business Administration, University of Illinois at Urbana-Champaign21780183

[B40] Hagedorn HJ Heideman PW The relationship between baseline Organizational Readiness to Change Assessment subscale scores and implementation of hepatitis prevention services in substance use disorders treatment clinics: a case study Implement Sci 2010 5 1 46 10.1186/1748-5908-5-46 20546584 PMC2902416

[B41] Yu W Ravelo A Wagner TH Phibbs CS Bhandari A Chen S Barnett PG Prevalence and Costs of Chronic Conditions in the VA Health Care System Med Care Res Rev 2003 60 3_suppl 146S 167 15095551 10.1177/1077558703257000

[B42] Kirchner JE Curran GM Aikens J Datapoints: detecting depression in VA primary care clinics Psychiatr Serv 2004 55 4 350 10.1176/appi.ps.55.4.350 15067144

[B43] Kauth MR Sullivan G Blevins D Cully JA Landes RD Said Q Teasdale TA Employing external facilitation to implement cognitive behavioral therapy in VA clinics: a pilot study Implement Sci 2010 5 10 75 20942951 10.1186/1748-5908-5-75 PMC2964555

[B44] Cohen J Statistical power analysis for the behavioral sciences 1988 Hillsdale, N.J.: L. Erlbaum Associates

[B45] Blow FC McCarthy JF Valenstein M Visnic S Gillon L Care for Veterans with Psychosis in the Veterans Health Administration, FY06 8th annual National Psychosis Registry 2007

[B46] Duncan E Dunlop BW Boshoven W Woolson SL Hamer RM Phillips LS Relative risk of glucose elevation during antipsychotic exposure in a Veterans Administration population Int Clin Psychopharmacol 2007 22 1 1 11 10.1097/01.yic.0000224794.29029.67 17159454

[B47] Lambert BL Cunningham FE Miller DR Dalack GW Hur K Diabetes risk associated with use of olanzapine, quetiapine, and risperidone in veterans health administration patients with schizophrenia Am J Epidemiol 2006 164 7 672 81 10.1093/aje/kwj289 16943266

[B48] Leslie DL Rosenheck RA Incidence of newly diagnosed diabetes attributable to atypical antipsychotic medications Am J Psychiatry 2004 161 9 1709 11 10.1176/appi.ajp.161.9.1709 15337666

[B49] Newcomer JW Second-generation (atypical) antipsychotics and metabolic effects: a comprehensive literature review CNS Drugs 2005 19 Suppl 1 1 93 15998156 10.2165/00023210-200519001-00001

[B50] Sernyak MJ Gulanski B Rosenheck R Undiagnosed hyperglycemia in patients treated with atypical antipsychotics J Clin Psychiatry 2005 66 11 1463 7 10.4088/JCP.v66n1117 16420085

[B51] Stroup TS Lieberman JA McEvoy JP Swartz MS Davis SM Rosenheck RA Perkins DO Keefe RS Davis CE Severe J Hsiao JK Effectiveness of olanzapine, quetiapine, risperidone, and ziprasidone in patients with chronic schizophrenia following discontinuation of a previous atypical antipsychotic Am J Psychiatry 2006 163 4 611 22 10.1176/appi.ajp.163.4.611 16585435

[B52] Consensus development conference on antipsychotic drugs and obesity and diabetes J Clin Psychiatry 2004 65 2 267 72 15003083 10.4088/jcp.v65n0219

[B53] Department of Veterans Affairs and Health Affairs DoD Washington DOoQPaPCS, Department of Defense VA/DoD Clinical Practice Guideline for the Diagnosis and Management of Dyslipidemia 2006

[B54] Department of Veterans Affairs D Washington DVEES, Offices of Quality & Performance and Patient Care Services, Department of Defense VA/DoD Clinical Practice Guideline for Screening and Management of Overweight and Obesity 2006

[B55] Marder SR Essock SM Miller AL Buchanan RW Casey DE Davis JM Kane JM Lieberman JA Schooler NR Covell N Stroup S Weissman EM Wirshing DA Hall CS Pogach L Pi-Sunyer X Bigger JT Jr Friedman A Kleinberg D Yevich SJ Davis B Shon S Physical health monitoring of patients with schizophrenia Am J Psychiatry 2004 161 8 1334 49 10.1176/appi.ajp.161.8.1334 15285957

[B56] Veterans Health Administration DoVAaHA, Department of Defense Washington DOoQaPp VA/DoD Clinical Practice Guideline for the Management of Diabetes Mellitus (DM) in Primary Care 2003

[B57] Veterans Health Administration DoVAaHA, Department of Defense VA/DoD Evidence-Based Clinical Practice Guideline Working Group. Washington DCOoQaPP Management of Persons with Psychoses 2004

[B58] Jennex A Gardner DM Monitoring and management of metabolic risk factors in outpatients taking antipsychotic drugs: a controlled study Can J Psychiatry 2008 53 1 34 42 18286870 10.1177/070674370805300106

[B59] Morrato EH Newcomer JW Allen RR Valuck RJ Prevalence of baseline serum glucose and lipid testing in users of second-generation antipsychotic drugs: a retrospective, population-based study of Medicaid claims data J Clin Psychiatry 2008 69 2 316 22 10.4088/JCP.v69n0219 18251625

[B60] Reid LD Lipid Profile Monitoring in Veterans Living with Schizophrenia-related disorders and treated with second-generation antipsychotics: Findings from a VA-based population 2007 10.1331/JAPhA.2008.07007 18595825

[B61] Weissman EM Zhu CW Schooler NR Goetz RR Essock SM Lipid monitoring in patients with schizophrenia prescribed second-generation antipsychotics J Clin Psychiatry 2006 67 9 1323 6 10.4088/JCP.v67n0901 17017817

[B62] Department of Veterans Affairs Office of the Inspector General Healthcare inspection: Atypical antipsychotic medications and diabetes screening and management 2007

[B63] Rubenstein LV Parker LE Meredith LS Altschuler A dePillis E Hernandez J Gordon NP Understanding team-based quality improvement for depression in primary care Health Serv Res 2002 37 4 1009 29 10.1034/j.1600-0560.2002.63.x 12236381 PMC1464007

[B64] Smith J Spollen J Owen R AcademyHealth: Orlando, FL Facilitation in implementing evidence-based practices for schizophrenia: Research and clinical leader perspectives 2007

[B65] Smith PC Kendall LM Hulin CL The measurement of satisfaction in work and retirement; a strategy for the study of attitudes 1969 Chicago, Ill.: Rand McNally20069516

[B66] Dhopesh VP Taylor KR Burke WM Survey of hepatitis B and C in addiction treatment unit Am J Drug Alcohol Abuse 2000 26 4 703 7 10.1081/ADA-100101903 11097200

[B67] Abraham HD Degli-Esposti S Marino L Seroprevalence of hepatitis C in a sample of middle class substance abusers J Addict Dis 1999 18 4 77 87 10.1300/J069v18n04_07 10631965

[B68] Hagedorn H Dieperink E Dingmann D Durfee J Ho SB Isenhart C Rettmann N Willenbring M Integrating hepatitis prevention services into a substance use disorder clinic Journal of Substance Abuse Treatment 2007 32 4 391 398 10.1016/j.jsat.2006.10.004 17481462

[B69] Almasio PL Amoroso P HAV infection in chronic liver disease: a rationale for vaccination Vaccine 2003 21 19-20 2238 41 10.1016/S0264-410X(03)00139-7 12744849

[B70] Reiss G Keeffe EB Review article: hepatitis vaccination in patients with chronic liver disease Aliment Pharmacol Ther 2004 19 7 715 27 10.1111/j.1365-2036.2004.01906.x 15043512

[B71] Stetler CB Legro MW Rycroft-Malone J Bowman C Curran G Guihan M Hagedorn H Pineros S Wallace CM Role of "external facilitation" in implementation of research findings: a qualitative evaluation of facilitation experiences in the Veterans Health Administration Implement Sci 2006 1 23 10.1186/1748-5908-1-23 17049080 PMC1635058

[B72] Streiner DL Norman GR Health measurement scales: a practical guide to their development and use 2003 Oxford; New York: Oxford University Press

[B73] Bernard HR Social Research Methods: Qualitative and Quantitative Approaches 2000 Thousand Oaks, CA: Sage

[B74] Schriesheim CA Powers KJ Scandura TA Gardiner CC Lankau MJ Improving construct measurement in management research: Comments and a quantitative approach for assessing the theoretical content adequacy of paper-and-pencil survey-type instruments Journal of Management 1993 19 2 385 417

[B75] Jöreskog KG Sörbom D LISREL 8: structural equation modeling with the SIMPLIS command language 1995 Chicago, Ill.; Hillsdale, N.J.: Scientific Software International; distributed by L. Erlbaum Associates

[B76] Haidet P Kelly PA Chou C Characterizing the patient-centeredness of hidden curricula in medical schools: development and validation of a new measure Acad Med 2005 80 1 44 50 10.1097/00001888-200501000-00012 15618092

[B77] Nagy M Using a single-item approach to measure facet job satisfaction Journal of Occupational and Organizational Psychology 2002 75 1 77 86 10.1348/096317902167658

[B78] Reckase MD The past and future of multidimensional item response theory Applied Psychological Measurement 1997 21 25 36 10.1177/0146621697211002

[B79] Molla A Licker PS Perceived e-readiness factors in e-commerce adoption: An empirical investigation in a developing country International Journal of Electronic Commerce 2005 10 1 83 110

[B80] Saldana L Chapman JE Henggeler SW Rowland MD The Organizational Readiness for Change scale in adolescent programs: Criterion validity Journal of Substance Abuse Treatment 2007 33 2 159 169 10.1016/j.jsat.2006.12.029 17434703 PMC2104560

[B81] Rampazzo L Angeli MD Serpelloni G Simpson DD Flynn PM Italian Survey of Organizational Functioning and Readiness for Change: A Cross-Cultural Transfer of Treatment Assessment Strategies European Addiction Research 2006 12 76 181 10.1159/000094419 16968992

