# Supplementary material for: Predicting implementation from organizational readiness for change: a study protocol
Source: Implement Sci. 2011 Jul 22;6:76. doi: 10.1186/1748-5908-6-76 (PMC3157428; doi:10.1186/1748-5908-6-76)
Supplement: Additional file 1 — Copy of the Organizational Readiness to Change Assessment instrument. This file is a PDF format of the Organizational Readiness to Change Assessment instrument with annotations about where the instrument is to be customized. [file 1748-5908-6-76-S1.PDF]

QUERI
Quality Enhancement Research Initiative

## *Organizational Readiness to Change Assessment*

## *Name of Station:* ________________________________________

## I. Evidence Assessment

**Finding: Practice goals in the VISN ## Cardiac Care Plan will improve outcomes for patients with acute coronary syndromes.**

Comment [Helfrich1]: This statement identifies the evidence-based practice or clinical change being evaluated.

Based on your assessment of the evidence basis for this statement, please rate the strength of the evidence in your opinion, on a scale of 1 to 5 where 1 is very weak evidence and 5 is very strong evidence:

| very weak | weak | neither weak nor strong | strong | very strong | Don't know / Not applicable |
|---|---|---|---|---|---|
| 1 | 2 | 3 | 4 | 5 | 99 |

Now, please rate the strength of the evidence basis for this statement based on how you think respected clinical experts in your institution feel about the strength of the evidence, on a 1 to 5 scale similar to the one above:

| very weak | weak | neither weak nor strong | strong | very strong | Don't know / Not applicable |
|---|---|---|---|---|---|
| 1 | 2 | 3 | 4 | 5 | 99 |

Staff discord over evidence

These items are intended to be differenced and assessed separately from the other evidence subscales.

For each of the following statements, please rate the strength of your agreement with the statement, from 1 (strongly disagree) to 5 (strongly agree)

The proposed practice changes or guideline implementation:

| | strongly disagree | disagree | neither agree nor disagree | agree | strongly agree | Don't know/ Not Applicable |
|---|---|---|---|---|---|---|
| a) are(is) supported by RCTs or other scientific evidence from the VA | 1 | 2 | 3 | 4 | 5 | 99 |
| b) are(is) supported by RCTs or other scientific evidence from other health care systems | 1 | 2 | 3 | 4 | 5 | 99 |
| c) should be effective, based on current scientific knowledge | 1 | 2 | 3 | 4 | 5 | 99 |
| ~~d) are(is) experimental, but may improve patient outcomes~~ | ~~1~~ | ~~2~~ | ~~3~~ | ~~4~~ | ~~5~~ | ~~99~~ |
| ~~e) likely won't make much difference in patient outcomes~~ | ~~1~~ | ~~2~~ | ~~3~~ | ~~4~~ | ~~5~~ | ~~99~~ |

Research evidence

These items were previously eliminated following subscale reliability analyses.

Practice experience

The proposed practice changes or guideline implementation:

| | strongly disagree | disagree | neither agree nor disagree | agree | strongly agree | Don't know/ Not Applicable |
|---|---|---|---|---|---|---|
| a) are supported by clinical experience with VA patients | 1 | 2 | 3 | 4 | 5 | 99 |
| b) are supported by clinical experience with patients in other health care systems | 1 | 2 | 3 | 4 | 5 | 99 |
| c) conform to the opinions of clinical experts in this setting | 1 | 2 | 3 | 4 | 5 | 99 |
| ~~d) have not been attempted in this clinical setting~~ | ~~1~~ | ~~2~~ | ~~3~~ | ~~4~~ | ~~5~~ | ~~99~~ |

This item was previously eliminated following subscale reliability analyses.

Patient needs

The proposed practice changes or guideline implementation:

| | strongly disagree | disagree | neither agree nor disagree | agree | strongly agree | Don't know/ Not Applicable |
|---|---|---|---|---|---|---|
| a) have been well-accepted by VA patients in a pilot study | 1 | 2 | 3 | 4 | 5 | 99 |
| b) are consistent with clinical practices that have been accepted by VA patients | 1 | 2 | 3 | 4 | 5 | 99 |
| c) take into consideration the needs and preferences of VA patients | 1 | 2 | 3 | 4 | 5 | 99 |
| d) appear to have more advantages than disadvantages for VA patients | 1 | 2 | 3 | 4 | 5 | 99 |

## II. Context Assessment

For each of the following statements, please rate the strength of your agreement with the statement, from 1 (strongly disagree) to 5 (strongly agree).

Leadership culture

Senior leadership/clinical management in your organization:

| | strongly disagree | disagree | neither agree nor disagree | agree | strongly agree | Don't know/ Not Applicable |
|---|---|---|---|---|---|---|
| a) reward clinical innovation and creativity to improve patient care | 1 | 2 | 3 | 4 | 5 | 99 |
| b) solicit opinions of clinical staff regarding decisions about patient care | 1 | 2 | 3 | 4 | 5 | 99 |
| c) seek ways to improve patient education and increase patient participation in treatment | 1 | 2 | 3 | 4 | 5 | 99 |

Staff culture

| Staff members in your organization: | strongly disagree | disagree | neither agree nor disagree | agree | strongly agree | Don't know/ Not Applicable |
|---|---|---|---|---|---|---|
| a) have a sense of personal responsibility for improving patient care and outcomes | 1 | 2 | 3 | 4 | 5 | 99 |
| b) cooperate to maintain and improve effectiveness of patient care | 1 | 2 | 3 | 4 | 5 | 99 |
| c) are willing to innovate and/or experiment to improve clinical procedures | 1 | 2 | 3 | 4 | 5 | 99 |
| d) are receptive to change in clinical processes | 1 | 2 | 3 | 4 | 5 | 99 |

Leadership practice

| Senior leadership/Clinical management in your organization: | strongly disagree | disagree | neither agree nor disagree | agree | strongly agree | Don't know/ Not Applicable |
|---|---|---|---|---|---|---|
| a) provide effective management for continuous improvement of patient care | 1 | 2 | 3 | 4 | 5 | 99 |
| b) clearly define areas of responsibility and authority for clinical managers and staff | 1 | 2 | 3 | 4 | 5 | 99 |
| c) promote team building to solve clinical care problems | 1 | 2 | 3 | 4 | 5 | 99 |
| d) promote communication among clinical services and units | 1 | 2 | 3 | 4 | 5 | 99 |

Evaluation/Accountability

| Senior Leadership/clinical management in your organization: | strongly disagree | disagree | Neither agree nor disagree | agree | strongly agree | Don't know/ Not Applicable |
|---|---|---|---|---|---|---|
| a) provide staff with information on VA performance measures and guidelines | 1 | 2 | 3 | 4 | 5 | 99 |
| b) establish clear goals for patient care processes and outcomes | 1 | 2 | 3 | 4 | 5 | 99 |
| c) provide staff members with feedback/data on effects of clinical decisions | 1 | 2 | 3 | 4 | 5 | 99 |
| d) hold staff members accountable for achieving results | 1 | 2 | 3 | 4 | 5 | 99 |

Opinion leader culture

| Opinion leaders in your organization: | strongly disagree | disagree | neither agree nor disagree | agree | strongly agree | Don't know/ Not Applicable |
|---|---|---|---|---|---|---|
| a) believe that the current practice patterns can be improved | 1 | 2 | 3 | 4 | 5 | 99 |
| b) encourage and support changes in practice patterns to improve patient care | 1 | 2 | 3 | 4 | 5 | 99 |
| c) are willing to try new clinical protocols | 1 | 2 | 3 | 4 | 5 | 99 |
| d) work cooperatively with senior leadership/clinical management to make appropriate changes | 1 | 2 | 3 | 4 | 5 | 99 |

Slack resources

| In general in my organization, when there is agreement that change needs to happen: | strongly disagree | disagree | neither agree nor disagree | agree | strongly agree | Don't know/ Not Applicable |
|---|---|---|---|---|---|---|
| a) we have the necessary support in terms of budget or financial resources | 1 | 2 | 3 | 4 | 5 | 99 |
| b) we have the necessary support in terms of training | 1 | 2 | 3 | 4 | 5 | 99 |
| c) we have the necessary support in terms of facilities | 1 | 2 | 3 | 4 | 5 | 99 |
| d) we have the necessary support in terms of staffing | 1 | 2 | 3 | 4 | 5 | 99 |

In a prior factor analyses, the slack resources subscale appeared to be a separate factor from other context subscales

## III. Facilitation Assessment:

For each of the following statements, please rate the strength of your agreement with the statement, from 1 (strongly disagree) to 5 (strongly agree):

Leadership roles in planning

| Senior leadership/clinical management will: | strongly disagree | disagree | neither agree nor disagree | agree | strongly agree | Don't know/ Not Applicable |
|---|---|---|---|---|---|---|
| a) propose a project that is appropriate and feasible | 1 | 2 | 3 | 4 | 5 | 99 |
| b) provide clear goals for improvement in patient care | 1 | 2 | 3 | 4 | 5 | 99 |
| c) establish a project schedule and deliverables | 1 | 2 | 3 | 4 | 5 | 99 |
| d) designate a clinical champion(s) for the project | 1 | 2 | 3 | 4 | 5 | 99 |

Project champion roles

| The Project Clinical Champion: | strongly disagree | disagree | neither agree nor disagree | agree | strongly agree | Don't know/ Not Applicable |
|---|---|---|---|---|---|---|
| a) accepts responsibility for the success of this project | 1 | 2 | 3 | 4 | 5 | 99 |
| b) has the authority to carry out the implementation | 1 | 2 | 3 | 4 | 5 | 99 |
| c) is considered a clinical opinion leader | 1 | 2 | 3 | 4 | 5 | 99 |
| d) works well with the intervention team and providers | 1 | 2 | 3 | 4 | 5 | 99 |

Leadership roles in support

| Senior Leadership/Clinical management/staff opinion leaders: | strongly disagree | disagree | neither agree nor disagree | agree | strongly agree | Don't know/ Not Applicable |
|---|---|---|---|---|---|---|
| a) agree on the goals for this intervention | 1 | 2 | 3 | 4 | 5 | 99 |
| b) will be informed and involved in the intervention | 1 | 2 | 3 | 4 | 5 | 99 |
| c) agree on adequate resources to accomplish the intervention | 1 | 2 | 3 | 4 | 5 | 99 |
| d) set a high priority on the success of the intervention | 1 | 2 | 3 | 4 | 5 | 99 |

Implementation team roles

| The implementation team members: | strongly disagree | disagree | neither agree nor disagree | agree | strongly agree | Don't know/ Not Applicable |
|---|---|---|---|---|---|---|
| a) share responsibility for the success of this project | 1 | 2 | 3 | 4 | 5 | 99 |
| b) have clearly defined roles and responsibilities | 1 | 2 | 3 | 4 | 5 | 99 |
| c) have release time or can accomplish intervention tasks within their regular work load | 1 | 2 | 3 | 4 | 5 | 99 |
| d) have staff support and other resources required for the project | 1 | 2 | 3 | 4 | 5 | 99 |

Implementation plan

| The implementation plan for this intervention: | strongly disagree | disagree | neither agree nor disagree | agree | strongly agree | Don't know/ Not Applicable |
|---|---|---|---|---|---|---|
| a) identifies specific roles and responsibilities | 1 | 2 | 3 | 4 | 5 | 99 |
| b) clearly describes tasks and timelines | 1 | 2 | 3 | 4 | 5 | 99 |
| c) includes appropriate provider/patient education | 1 | 2 | 3 | 4 | 5 | 99 |
| d) acknowledges staff input and opinions | 1 | 2 | 3 | 4 | 5 | 99 |

Communication

| Communication will be maintained through: | strongly disagree | disagree | neither agree nor disagree | agree | strongly agree | Don't know/ Not Applicable |
|---|---|---|---|---|---|---|
| a) regular project meetings with the project champion and team members | 1 | 2 | 3 | 4 | 5 | 99 |
| b) involvement of quality management staff in project planning and implementation | 1 | 2 | 3 | 4 | 5 | 99 |
| c) regular feedback to clinical management on progress of project activities and resource needs | 1 | 2 | 3 | 4 | 5 | 99 |
| d) regular feedback to clinicians on effects of practice changes on patient care/outcomes | 1 | 2 | 3 | 4 | 5 | 99 |

Assessment

| Progress of the project will be measured by: | strongly disagree | disagree | neither agree nor disagree | agree | strongly agree | Don't know/ Not Applicable |
|---|---|---|---|---|---|---|
| a) collecting feedback from patients regarding proposed/implemented changes | 1 | 2 | 3 | 4 | 5 | 99 |
| b) collecting feedback from staff regarding proposed/implemented changes | 1 | 2 | 3 | 4 | 5 | 99 |
| c) developing and distributing regular performance measures to clinical staff | 1 | 2 | 3 | 4 | 5 | 99 |
| d) providing a forum for presentation/discussion of results and implications for continued improvements | 1 | 2 | 3 | 4 | 5 | 99 |

Project resources

| The following are available to make the selected plan work: | strongly disagree | disagree | neither agree nor disagree | agree | strongly agree | Don't know/ Not Applicable |
|---|---|---|---|---|---|---|
| a) staff incentives | 1 | 2 | 3 | 4 | 5 | 99 |
| b) equipment and materials | 1 | 2 | 3 | 4 | 5 | 99 |
| c) patient awareness/need | 1 | 2 | 3 | 4 | 5 | 99 |
| d) provider buy-in | 1 | 2 | 3 | 4 | 5 | 99 |
| e) intervention team | 1 | 2 | 3 | 4 | 5 | 99 |
| f) evaluation protocol | 1 | 2 | 3 | 4 | 5 | 99 |

Evaluation

| Plans for evaluation and improvement of this intervention include: | strongly disagree | disagree | neither agree nor disagree | agree | strongly agree | Don't know/ Not Applicable |
|---|---|---|---|---|---|---|
| a) periodic outcome measurement | 1 | 2 | 3 | 4 | 5 | 99 |
| b) staff participation/satisfaction survey | 1 | 2 | 3 | 4 | 5 | 99 |
| c) patient satisfaction survey | 1 | 2 | 3 | 4 | 5 | 99 |
| d) dissemination plan for performance measures | 1 | 2 | 3 | 4 | 5 | 99 |
| e) review of results by clinical leadership | 1 | 2 | 3 | 4 | 5 | 99 |

*The Organizational Readiness to Change Assessment (ORCA) was developed by the Ischemic Heart Disease Quality Enhancement Research Initiative of the Veterans Health Administration. For more information contact Christian Helfrich, christian.helfrich@va.gov or 206-277-1655.*
