# Supplementary material for: Predicting implementation from organizational readiness for change: a study protocol
Source: Implement Sci. 2011 Jul 22;6:76. doi: 10.1186/1748-5908-6-76 (PMC3157428; doi:10.1186/1748-5908-6-76)
Supplement: Additional file 2 — Description of four partner projects. This file is a PDF document describing each of the four partner projects contributing data to the study for the described protocol, including the project aims, methods and details about the use of the ORCA. [file 1748-5908-6-76-S2.DOC]

**Additional File 2: Description of the four partner projects**

**Partner projects**

Each of the four partner projects are quality-improvement intervention studies that test the effects of an external facilitation intervention on the implementation of an evidence-based practice. External facilitation is what Stetler and colleagues have described as a process of interactive problem-solving and support [71]. No single strategy is employed; rather, external facilitation requires flexibility and a variety of techniques and approaches that evolve and are responsive to variable site characteristics, resources, and barriers. External facilitation is based on concepts put forth in the PARIHS model. Below we describe each of the partner projects.

**Partner Project #1: Evaluating Facilitation of CBT Implementation in VA Primary Care**

The first partner project is the Evaluating Facilitation of Cognitive Behavioral Therapy Implementation in VA Primary Care study (Dean Blevins, PhD-PI, Veterans Health Administration (VHA) grant #RRP 08-239) at the Central Arkansas Veterans Healthcare System. It was conducted from October 2008 to March 2009. The purpose was to compare implementation of cognitive behavioral therapy (CBT) for depression, with and without external facilitation.

Depression is a high incidence, high burden condition in VHA [41-42], and CBT is a very effective treatment though often not available in VA clinics. Mental health providers in primary care settings in one VA network (Veterans Integrated Service Network (VISN) 16) were trained in CBT in May, 2008. This group included 40 providers in 10 VA medical centers (VAMCs) and 10 community-based outpatient clinics (CBOCs). Half of these were randomly selected to receive external facilitation following CBT training for a period of six months. External facilitation involved an initial face-to-face meeting between the facilitator and facilitated therapists, individual goal setting, monthly conference calls, and frequent email and telephone correspondence to monitor goal progress and engage in problem solving to increase implementation. The remaining 10 sites served as matched control sites, receiving CBT training but no external facilitation. Sites were matched according to the number of unique veterans treated, facility type (VAMC or CBOC), and leadership buy-in and interest in implementing CBT. The ORCA was fielded among each site’s mental health providers (40 individuals at 20 sites) at the end of September 2008, i.e., four months following the CBT training, and 3 months after initiation of the external facilitation intervention. Surveys were returned by 26 of 40 providers from 18 of 20 facilities. The ORCA response rate was 65% for providers and 90% for facilities. Fewer than 1% of items in returned surveys had missing values.

The dependent variable was self-reported percent clinical time spent conducting CBT in the past 30 days [43], assessed at baseline, just before the CBT workshop, and at 6 months. The ORCA was assessed at the beginning of month 4, halfway through the facilitation intervention.

For the analyses, we treat the ORCA assessment at 4 months as a baseline assessment, because experience with the external facilitation intervention suggests that implementation activities were likely still in development during that period and new patterns of practice would not have been well established. The partner project also collected data on the total number of psychotherapy encounters and total number of psychotherapy unique encounters to control for facility size. Implementation outcome will be calculated as the difference in self-reported CBT use at follow-up minus use at baseline, expressed as a proportion (numerator = difference score; denominator = possible change in practice [maximum possible difference from baseline to follow-up]). This proportion will be converted to Cohen’s h, an effect size [44].

**Partner project #2: Monitoring and Management for Metabolic Side Effects of Antipsychotics**

The second partner project is the Monitoring and Management for Metabolic Side Effects of Antipsychotics study (Richard Owen, MD-PI, VHA grant #SDP 08-375-2). It tests an intervention to augment a national implementation strategy to improve monitoring of metabolic side effects of antipsychotics. Baseline ORCA measures will occur June-August 2011 and follow-up ORCA will be collected June-August 2012.

The preferred treatment for psychotic disorders is second-generation antipsychotics (SGAs) [45]. Though safe and effective, SGAs often produce adverse metabolic side effects such as obesity and diabetes [46-51], and evidence-based treatment recommendations now include monitoring and management of these side effects [52-57]. However, research finds low rates of metabolic monitoring and delays in management of metabolic abnormalities among patients treated with antipsychotics [50, 58-61]. A recent report by the VA Office of the Inspector General called for timelier monitoring and management of antipsychotics’ metabolic side effects [62]. This partner project will test an implementation intervention to enhance uptake of evidence-based tools and strategies to improve monitoring and management of metabolic side effects of antipsychotics, concurrently with a planned VA national effort to implement evidence-based practices for monitoring and management. The study includes an experimental intervention with some sites randomized to receive external facilitation.

The 3-year study will have four contiguous phases: pre-implementation (12 months), evidence-based quality improvement with facilitation (EBQI/F) (6 months), and sustainability (6 months). The study has three primary aims: 1) to test the effect of the EBQI/F intervention on monitoring of metabolic side effects of antipsychotics in sites likely to have fewer resources for quality improvement and greater challenges to implementation; 2) to test the effect of the EBQI/F intervention on management of metabolic side effects of antipsychotics in participating sites; and 3) to assess the direct costs of the EBQI/F intervention and to explore potential variations in costs of the intervention in sites with low- versus high- organizational readiness-to-change. The EBQI/F intervention combines (a) EBQI processes in the pre-implementation phase to design and tailor the initial implementation strategy to local context in a manner consistent with available research evidence [63], and (b) external facilitation to support, monitor, problem-solve and refine implementation, as needed, during the study’s implementation phase [71]. Planned external facilitation activities in the project are consistent with key components of external facilitation identified in previous research [71], and has been successfully used in recent studies by the partner project team [64].

The study will be conducted at sites with likely implementation challenges including insufficient staffing, provider and staff stress, few resources to conduct QI efforts; and which are performing poorly on indicators of metabolic monitoring and management at the time of site selection.

During study start up, twelve sites will be selected for study inclusion. At the start of the EBQI phase (June-August 2011), the ORCA and Job Satisfaction Index (JSI) [65] will be administered as a baseline assessment with providers and each Mental Health Service Chief per site (estimated 10 per site). The ORCA and JSI will be administered again at the conclusion of the sustainability phase (June-August 2012). The implementation outcome will be compliance with guidelines for monitoring and management of metabolic side effects to antipsychotics, calculated as the proportion of patients receiving guideline-concordant metabolic side effect monitoring and management. Implementation outcomes will be collected monthly, beginning at baseline. Baseline data will be available by October 2011.

**Partner project #3:**  **Promoting Implementation of MyHealth*e*Vet among Veterans with Spinal Cord Injuries and Disorders (SCI/D)** The third partner project is the Promoting Implementation of MyHealth*e*Vet among Veterans with SCI/D study (Timothy P. Hogan, PhD-PI, VHA grant #RRP 09-129). It tested an intervention to promote use of the MyHealtheVet (MHV) system among veterans with SCI/D and their healthcare providers. MHV is an integrated Web portal and personal health record (PHR). The study assessed the effectiveness of two promotional campaign strategies for implementing MHV: 1) a basic facilitation strategy and 2) an enhanced facilitation strategy.

The objective of the interventions was to increase the proportion of veterans who complete an identity verification process referred to as “in-person authentication” (IPA), which allows them to use the most sophisticated components of the MHV system, including the ability to view upcoming appointments and customized reminders for preventative care, and to exchange secure messages with healthcare team members. Anecdotal evidence suggested that the process for completing IPA, which involves a series of orientation activities and visiting a VA facility to verify one’s identity, posed a barrier to MHV utilization.,

The MHV study involved a multifaceted, two-phase design. In Phase One, as part of a broader formative evaluation effort that involved semi-structured interviews with healthcare providers, a mail survey of Veterans, and corresponding chart reviews, the study team used the ORCA to evaluate healthcare provider perceptions of evidence and contextual factors that influence use of PHR systems like MHV. Phase One findings were used to inform promotional campaigns intended to encourage activation of veterans with SCI/D and VA SCI/D healthcare providers, engage them with the MHV system, and advance system uptake. In Phase Two, the two study sites implemented the promotional campaigns based on the findings from Phase One; one site received enhanced facilitation and the other basic facilitation.

Basic facilitation comprised three elements: 1) mailing of MHV introductory materials to veterans on the SCI/D registry at the site; 2) a MHV orientation session for VA SCI/D healthcare providers; and 3) onsite distribution and display of MHV promotional materials. The enhanced facilitation site included the aforementioned basic facilitation elements as well as: 1) MHV Clinician Coaching, which involved the distribution of MHV orientation materials developed specifically for providers, system demonstration, and question and answer sessions held as part of regularly scheduled administrative meetings; 2) MHV Veteran Training, which involved incorporating MHV as a topic in an existing patient education series, the dissemination of MHV information in conjunction with other planned patient activities (i.e., recreational outings, athletic events) and the facility’s homecare program, and hands-on system orientation and training in a facility computing lab; and 3) IPA Process Support, which involved an analysis of organizational processes, barriers, and “bridge-building” opportunities between the SSCI Center and other facility units involved in IPA. Support for completing IPA was provided to outpatients as part of routine appointments as well as to inpatients. A staff member (i.e., social worker), appointed by the SCI Chief, was trained on MHV by the study team and served as a MHV champion for the SCI Center over the course of the intervention period. The champion helped to design the enhanced facilitation activities and enlisted several colleagues to help her conduct them.

The ORCA was fielded at baseline among providers and staff identified by the project point of contact at each study site, and the outcome, % of veterans who completed IPA, was assessed at 6 months. The implementation outcome of the project was the proportion of veterans with IPA reported as a site-level effect size. The ORCA was fielded to 26 participants with 25 returning completed surveys (96% response rate); 16 received the entire ORCA; 10 received just the Evidence and Context scales due to initial confusion over answering the Facilitation items; at baseline, respondents did not feel that they had enough experience with implementing the facilitation-based promotional campaigns to provide adequate responses to the question items.

**Partner project #4:** Evaluation of Educational Programs for Hepatitis C Care

The fourth partner project is the Evaluation of Educational Programs for Hepatitis C Care study (Hildi Hagedorn, PhD-PI). It is a quality improvement project sponsored by the VA Substance Use Disorder Quality Enhancement Research Initiative (SUD-QUERI) and the VA Minneapolis Hepatitis C Resource Center to improve hepatitis testing, education, prevention, and referral services in substance use disorder clinics. The intervention involves education and training of clinician teams from each participating clinic and was conducted in two cohorts: the first one (7 clinics in VISN 23) in June 2009 (respondents=23, facilities=7) and the second (18 clinics) in September 2010 (respondents=42, facilities=18). The project has been reviewed and exempted by the VA Minneapolis IRB (IRB # 03687-A). The project is led by Dr. Hildi Hagedorn, a clinical psychologist and Implementation Research Coordinator for the SUD-QUERI.

Rates of hepatitis C infection in drug and alcohol dependent individuals has been shown to be much higher than the general population rate of less than two percent. [66-67] Dr. Hagedorn's evaluation in the Minneapolis VA Addictive Disorders Service reported a rate of 12.4%.[68] Because of common risk factors, individuals at high risk for hepatitis C are also at higher risk for exposure to hepatitis A and B, both vaccine preventable diseases. In addition, patients already compromised by chronic liver disease, due to either hepatitis C or alcoholic liver disease are at a much higher risk for poor outcomes including fatality if co-infected with acute hepatitis A and B.[69-70] Therefore, substance use disorders clinics are the ideal location to provide preventive services, including education and vaccinations, and also for identification of individuals already infected with chronic hepatitis infections so that they can be connected to treatment services.

Dr. Hagedorn’s quality improvement project will test an implementation intervention consisting of three phases. Phase 1: Participating teams completed a pre-training needs assessment detailing current hepatitis services provided by the clinic. Phase 2: Participating teams attended a 2-day in-person training including education on hepatitis viruses and their prevalence in individuals who abuse substance, education on implementation strategies, and development of an action plan. The action plan compared the teams’ reported baseline services to service recommendations allowing identification of areas for improvement. The teams selected specific quality improvement goals, described initial steps toward those goals which can be completed within one month of the training, and designated the persons responsible for each goal. In the second cohort (SUD), the in-person training was followed by six months of facilitation phone calls between a member of the training staff and the site's implementation team. Prior outcome evaluations of this program have demonstrated that it was effective at promoting implementation of two or more new hepatitis services in 82% of training teams.

Dr. Hagedorn’s project included two-cohorts of implementation teams (7 medical centers in VISN 23; 18 medical centers from across the country that volunteered to participate). Each team was encouraged to include a person in a leadership role from the addictive disorders team or the mental health service line, a clinical staff person from the addictive disorders team with a specific interest in implementation of services for prevention of infectious diseases, and a representative from the gastroenterology clinic. Needs assessments were sent out in May, 2009 for the first cohort and in August 2010 for the second cohort. The in-person training took place June 11^th^ and 12^th^, 2009 (VISN 23 cohort) and September 15^th^ and 16^th^, 2010 (second cohort). Facilitation calls for the first cohort took place from July 2009 through December 2009. Facilitation calls for the second cohort took place from October 2010 through March 2011. At baseline and at six-month follow-up, clinic teams completed a survey assessing current testing, education, vaccination, and referral services available. Implementation success was determined based on the number of new services established at the clinic (e.g., two or more new services indicates high level of change).

At the start of both project cohorts (June 2009 and September 2010), the project team fielded the evidence and context scales of the ORCA and the Job Satisfaction Index (JSI) as a baseline assessment with the participating clinician teams. For cohort one, clinician teams were asked to distribute the ORCA to the substance abuse clinic staff at their site. The full ORCA was administered again at the conclusion of the second cohort (April 2011). For cohort #1, the ORCA response rate could not be calculated, as the program attendees distributed the ORCA to all staff in their clinics and did not record the number of surveys distributed . For cohort #2, completion of the baseline ORCA was a prerequisite to receiving a CE certificate for the program, and the response rate was 100% at baseline and 60% (25/42) at follow-up. The implementation outcome measures were self-reported use of screening, treatment, and education procedures. Implementation outcomes were collected via survey at baseline and at follow-up.

**Inclusion of women and minorities**

A brief note about the composition of the study population in terms of representation of sex/gender and racial/ethnic groups. All data are secondary data collected by partner projects according to their research protocols; respondent demographic data are not being collected and are therefore unavailable to the proposed study. Measurement equivalence among respondents of different sex/gender and/or racial/ethnic groups will be an important topic for future research.
